# Letter to the Editor regarding: “History of dermatology: the study of skin diseases over the centuries”^☆^^☆☆^

**DOI:** 10.1016/j.abd.2021.06.001

**Published:** 2021-07-21

**Authors:** Mohammad Reza Mirzaei, Javad Ghazi-Sha’rbaf, Reza Mohammadinasab

**Affiliations:** a Department of Traditional Medicine, School of Traditional Medicine, Tabriz University of Medical Sciences, Tabriz, Iran; b Department of Islamic History and Civilization, Faculty of Theology, Azarbaijan Shahid Madani University, Tabriz, Iran; c Department of History of Medicine, School of Traditional Medicine, Tabriz University of Medical Sciences, Tabriz, Iran

Dear Editor,

We passionately studied the article named “History of dermatology: the study of skin diseases over the centuries”.^1^ In our view, this is an outstanding manuscript that will be acclaimed by scholars and readers.

This article reviews and inquires about skin diseases over the centuries. This is a comprehensive study with very detailed and useful tips in which the authors have reviewed various works. However, it also has an important drawback as the authors did not refer to the medical tradition of skin diseases in medieval Islamic countries. The medieval medicine in the Islamic world, which began with the movement to translate Greek, Iranian, and Indian works, could flourish by the arrival of individuals such as Rhazes, Haly Abbas and Avicenna.^2^, ^3^ Each of the important physicians of the era allocated chapter(s) of their works to types, causes, symptoms, and treatment of skin, hair, and nail diseases, including psoriasis, warts, vitiligo, smallpox, blemishes, and leprosy. Avicenna’s The Canon of Medicine and Rhazes’s Al-Hawi were examples of many works with detailed discussions in separate chapters.^4^, ^5^, ^6^ For instance, Avicenna allocated separate chapters of the Canon of Medicine to skin diseases, hair diseases, and skin color diseases. Furthermore, he considered a chapter in this book for makeup in which he described nail diseases and issues relating to makeup and beauty, as well as fitness in detail.^5^, ^6^

We hope that this explanation has been able to show the course of the history of medicine in the Middle Ages, its importance, and also to fill the gap in the article.

## Financial support

None declared.

## Authors' contributions

Mohammad Reza Mirzaei: Approval of the final version of the manuscript; design and planning of the study; drafting and editing of the manuscript.

Javad Ghazi-Sha’rbaf: Approval of the final version of the manuscript; design and planning of the study; drafting and editing of the manuscript.

Reza Mohammadinasab: Approval of the final version of the manuscript; design and planning of the study; drafting and editing of the manuscript.

## Conflicts of interest

None declared.
